# The Distressing Nervous Symptoms in an Habitual Smoker, Which Resulted from His Abstaining from the Use of Tobacco

**Published:** 1860-07

**Authors:** J. Levison

**Affiliations:** London.


### AMERICAN JOURNAL

OF

### DENTAL SCIENCE.

Vol. XI. NEW SERIES.—JULY, 1860. No. 3.

### ORIGINAL COMMUNICATIONS.

### ARTICLE I.

*The Distressing Nervous Symptoms in an Habitual Smoker, which resulted from his Abstaining from the Use of Tobacco.* By Dr. J. Levison, of London.

*To the Editors,*

*Sirs:*

Some years since the writer of this article published a paper in the "Medical Times," on the Diseases of the Fangs Peculiar to Smokers; in which it was shown that this disease was confined in a majority of instances, to the destruction of the periosteum; and that when the roots thus deprived both of protection and nutriment, that they gradually suffer from absorption at their extremities, that ultimately the fangs are merely extraneous bodies, and as a consequence, great local irritation followed. That in this "smoker's disease," there was induced the most agonizing suffering, similar in kind to acute *tic douloureaux.*

But the following case shows that there may result many morbid symptoms by abstaining from the use of tobacco after a habitual practice of many years standing ; and yet that it would be most wise and thrifty not to render necessary for the system a poison so deadly in its nature.

Without any further preface we will state the case as one worthy of the consideration both of the physiologist and pathologist. We may state that the sufferer was a professional man, a native of Essex, (a county in England,) that his father was a German and his mother English. From boyhood he had been used to the society of learned foreigners, who invariably discussed politics, religion, philosophy, literature and the arts, with their odorous meerschaums, so that from mere imitation he acquired an early inclination for the Indian weed, and actually commenced the habit at the age of thirteen.

Years rolled on—he had passed through his novitiate, had been a great traveler, and ultimately settled down a practitioner of surgery. He married and had a rather large family, and during all these years he had continued to smoke cigars or pipes, (when not engaged in his profession,) but invariably so when either reading or writing. This routine practice he continued until the age of thirty-seven, when owing to the circumstance of having a female relative on a visit to him, who after returning to his house from a walk, became so affected with the fragrant fumes of his pipe, as to cough most violently for a very long time ; this so annoyed him that he vowed that he would cease to indulge in such a selfish practice, if by so doing he must cause pain, and thus unwittingly injure any one. And with a magnanimity our smoker smashed his pipe, saying, "now I have broken the spell, and shall henceforth renounce the use of tobacco, with all its soothing pleasures for ever." Poor fellow, he knew not what he vowed, or what tortures he would inflict on himself as the consequence of this rashness.

He was in his habits a teetotaler, although not a pledged one ; and was *very* fat, having the outward and visible sign of an extremely nervous temperament.

"A lean and hungry look."

Not long after his anti-smoking determination, he suffered from a most curious and painful annoyance, an almost constant noise in his head, which described a booming sound, like the rush of mighty waters, or as if he had been placed between the Pacific and Atlantic oceans, and heard the hollow sounds of the rolling waves. And so far as to the fact of the noise, it was real and not imaginary, for whenever he laid his head on the pillow, his wife could hear it in the same manner as may be heard in the silent hour of night the audible sounds of a heart strongly palpitating.

From his own knowledge he was aware that these sounds arose from too active vascular circulation, but he never entertained the idea what was the predisposing cause. He had, as he related, the advantage of a multitude of medical counsellors, but though he admitted that they were wise in their generation, yet in his special instance they did not manifest their usual sagacity.

In proof of this, he cited among other evidence, that he sent to consult an M. D., who had devoted himself to affections of the brain, and of the nervous system generally, when the following conversation took place, which we report without comment.

Patient.—"My dear doctor, I have come to consult you, and accordingly he detailed the disagreeable symptoms already mentioned, and then added, "That besides the noise in his head, he had some horrid morbid affection of the sphincter muscles of the rectum, which was so agonizing when passing fecal matter, that he often fainted away when performing this necessary act."

Doctor.—"I am sorry for you my dear fellow, but it is evident that you have an incipient inflammation of the membranes of the brain, and will become either raving mad or an idiot."

Patient (somewhat irritated,) said, "Sir, you either compliment the present state of my intellect, or else you are a very injudicious physician. For, to an ordinary person, your opinion would be regarded as a verdict, so that your prediction would very probably in such a case become verified. But I do not think you understand my affection."

The doctor also colored up, somewhat piqued, which his new patient observed, and as they had been old friends, he thus qualified his commentary and soothed the vanity of the medical judge.

PATIENT.—Pray dont think my remarks were intended to be rude or discourteous; and if you will allow me more fully to explain myself, I feel you will acquit me of any such intention. If there were any organic lesion my sufferings would be constant, but now there are periods of intermission. And I have noticed that too much anxiety or application to mental pursuits, or a hearty dinner, invariably brings on an attack of greater severity than in a more normal state of my health."

The doctor expressed his sympathy and recommended the patient to travel. He did so, and yet he continued to endure these two sources of annoyance for more than two years longer, (the noise in the head and the affection of the rectum,) and he has declared since that he wondered he did not commit suicide. Nothing he said saved him but a strong religious sentiment, and the necessity of attending to his professional duties. Yet he never once attributed his sufferings from his having abstained from the use of tobacco.

At length the morbid affection of the rectum increased in intensity, and this induced him to apply to the late Mr. Alfred Jukes, of Birmingham, (a most excellent surgeon and most worthy man,) who had just published a monograph on "Diseases of the Rectum," to whom he related his symptoms. The worthy gentleman expressed his sorrow, as he personally esteemed the patient, and recommended him to take a grain of opium twice a day, and to use as an injection, an infusion of poppies.

On leaving Mr. Jukes, he says, "that he walked away quite melancholy, feeling that if he acted on this advice, he must ultimately become an opium drunkard, as he knew from experience that he would have to increase the dose from time to time. Fortunately for himself, he was a man who was in the habit of reasoning on all phenomena, and to inquire into their special causes. Therefore, when his friend ordered opium, he asked himself a few questions: If, said he, "all my sufferings have resulted from my constitution requiring a narcotic, then it is very probable that all my torture for the past years has been induced from depriving my system of the narcotizing influence of tobacco? If so, (he added as a corollary,) "I'll take a choice of evils, and resume smoking." He did so, and found some difficulty in the attempt. Fortunately for him, just about the time he was endeavoring to recommence the habit, a friend of his had sent from America, a box of very fine Havanas, for his use; yet he had some difficulty to consume a half a cigar, but he persevered, and it is now more than twenty years since, and he has never had the least return of the noise in his head, or the painful affection of the rectum, and of course continued to narcotize himself with tobacco. But he has paid the penalty of losing many teeth, without the slightest caries in the crowns, but always with the periosteum denuded from the fangs, which are as rough at their extremities, as if rasped.

He nevertheless deprecates the habit of smoking, from the latter consequence, and would never recommend any one to commence the practice. It seems certainly unwise to use any means to render the system in such an abnormal condition to require a deadly poison; but on the other hand, when the habit becomes constant, so as to assume a chronic form, then it seems very injudicious to recommend its discontinuance altogether. For if by so doing such morbid systems, as described in the case now reported for the first time do not occur, yet others may, which may also induce some form of irritability.

